# DNA Subunit Vaccine and Recombinant BCG Based on Mycobacterial Lipoprotein LprO Enhance Anti-Tuberculosis Protection in the Lungs of Mice

**DOI:** 10.3390/vaccines13040400

**Published:** 2025-04-11

**Authors:** Weili Huang, Shuqin Xu, Lifang Shen, Dan Chen, Hanmei Liu, Yuting Tang, Xiaolin Liu, Wenxuan Xiao, Ziwei Zhou, Shifeng Zhang, Jixi Li, Xiaoyong Fan, Yuefeng Chu, Lu Zhang

**Affiliations:** 1 State Key Laboratory of Genetics and Development of Complex Phenotypes, Department of Microbiology, School of Life Sciences, Fudan University, Shanghai 200438, China; 24110700173@m.fudan.edu.cn (W.H.); 19210700031@fudan.edu.cn (S.X.); 15110700036@fudan.edu.cn (L.S.); 20110700150@fudan.edu.cn (D.C.); 19210700021@fudan.edu.cn (H.L.); 20210700030@fudan.edu.cn (Y.T.); 22110700144@m.fudan.edu.cn (W.X.); 18210700053@fudan.edu.cn (Z.Z.); 22210700073@m.fudan.edu.cn (S.Z.); lijixi@fudan.edu.cn (J.L.); 2 National Heart & Lung Institute, Imperial College London, London SW3 6LY, UK; xiaolin.liu24@imperial.ac.uk; 3 Shanghai Institute of Infectious Diseases and Biosecurity & Shanghai Public Health Clinical Center, Fudan University, Shanghai 201508, China; xyfan008@fudan.edu.cn; 4 State Key Laboratory for Animal Disease Control and Prevention, College of Veterinary Medicine, Lanzhou Veterinary Research Institute, Lanzhou University, Chinese Academy of Agricultural Sciences, Lanzhou 730046, China; 5 Shanghai Engineering Research Center of Industrial Microorganisms, Shanghai 200438, China; 6 MOE Engineering Research Center of Gene Technology, Shanghai 200438, China

**Keywords:** tuberculosis, lipoprotein antigens, rBCG-lprO, DNA-lprO vaccine

## Abstract

**Background/Objectives**: Over the past two centuries, tuberculosis (TB) has been responsible for approximately one billion deaths and continues to represent a significant global health challenge. Despite extensive research efforts, fully effective strategies for the prevention or eradication of TB remain elusive, highlighting the urgent demand for novel vaccines with enhanced safety profiles and efficacy. Lipoproteins, integral surface proteins of mycobacteria, are frequently associated with virulence and display notable immunogenicity, rendering them promising candidates for vaccine development. This study investigates the potential of the mycobacterial lipoprotein, LprO, as a vaccine antigen against TB. **Methods**: A pcDNA-*lprO* DNA vaccine was constructed, and its immunogenicity was evaluated using a murine model. Its protective efficacy was further assessed using a *Mycobacterium marinum* (*M. marinum*)-infected zebrafish model. Additionally, a recombinant BCG vaccine strain, BCG Japan::pNBV1-lprO, was generated. Its immunogenicity was tested in mice, and its safety was evaluated in SCID mice. Both vaccine candidates were further assessed in regard to their protective efficacy in a murine *Mycobacterium tuberculosis* (M. tb) infection model. **Results**: The pcDNA-*lprO* vaccine increased the M. tb-specific IFN-γ-secreting lymphocytes in murine spleens and prolonged the survival of zebrafish infected with *M. marinum*. The recombinant BCG Japan::pNBV1-*lprO* vaccine elicited M. tb-specific Th1-type immune responses in mice compared to the standard BCG Japan strain. Both vaccines effectively reduced the bacterial burden of M. tb in murine lungs, offering superior protection relative to the control groups. **Conclusions**: These findings establish LprO as a compelling candidate for TB vaccine development, with both LprO-based DNA and recombinant BCG vaccines demonstrating robust protective effects against TB.

## 1. Introduction

Tuberculosis (TB) is a severe respiratory infectious disease caused by the pathogen *Mycobacterium tuberculosis* (M. tb). It remains the second leading cause of mortality from a single infectious agent worldwide [1]. Presently, the Bacillus Calmette–Guérin (BCG) vaccine is the only licensed vaccine for TB prevention. However, its efficacy is limited, providing protection for only 10–20 years and presenting notable safety concerns for immunocompromised individuals [2,3]. Consequently, there is an imperative need for the development of vaccines that ensure enhanced safety and improved protective efficacy.

In mycobacteria, lipoproteins, situated within the bacterial plasma membrane or outer membrane, serve as crucial surface proteins constituting the bacterial envelope [4]. During infection, these lipoproteins facilitate adhesion, colonization, evasion of the host immune system, and modulation of immune responses [5]. Approximately 99 lipoproteins have been identified in M. tb, each fulfilling specific functional roles [5]. Among these, LppX is notable; the deletion of *lppX* diminishes M. tb’s virulence, a reduction attributed to a unique hydrophobic cavity within its protein structure that mediates the transport of phthiocerol dimycocerosates (PDIMs) to the outer cell membrane [6,7]. Another significant lipoprotein, RpfB, one of the resuscitation-promoting factors, facilitates M. tb’s reactivation from dormancy by cleaving the glycosidic bond between N-acetylglucosamine and N-acetylmuramic acid, thereby compromising cell wall integrity [8]. Certain lipoproteins, such as LpqH, directly influence the host immune system. LpqH downregulates MHC class II expression, aiding M. tb’s immune evasion, and disrupts macrophage cytokine production [9].

In recent years, lipoproteins with diverse functions have been explored as potential vaccine candidates against M. tb, capitalizing on their immunological properties (Table 1). Many lipoprotein-based vaccines have demonstrated considerable efficacy in combating M. tb. For instance, the M. tbΔ*lpqS* attenuated live vaccine exhibits comparable effectiveness to BCG in guinea pig models [10]. In mouse models, the RpfB protein vaccine reduces the bacterial load in the lungs and spleens by 2 log_10_ and 1 log_10_, respectively [11]. Similarly, the PstS-3 DNA vaccine achieves a 1.5 log_10_ reduction in the lung bacterial burden in mice [9]. Collectively, these findings position M. tb lipoproteins as promising antigens for anti-M. tb vaccine development.

LprO (possible lipoprotein LprO, Rv0179c) is one such lipoprotein, found predominantly in pathogenic mycobacteria. In this study, LprO was utilized as an antigen to construct two vaccine types: the pcDNA-*lprO* DNA vaccine and the BCG Japan::pNBV1-*lprO* live vaccine. Acting as a vaccine antigen, LprO amplifies Th1-type immune responses, leading to a reduction in the M. tb burden in murine lungs. These findings suggest that LprO could be a valuable antigen for M. tb, presenting a novel target for vaccine research and a potential strategy for tuberculosis prevention.

## 2. Methods and Materials

### 2.1. Prediction of LprO Epitopes

To predict CD4+ T cell epitopes for the target gene, the NetMHC II 2.3 Server (http://www.cbs.dtu.dk/services/NetMHCII/, accessed on 8 April 2025) was utilized. The FASTA file containing the amino acid sequence of the gene (retrievable from the NCBI website: https://www.ncbi.nlm.nih.gov/gene/886796, accessed on 8 April 2025) was imported. The amino acid length was set to 15, and the IC50 threshold was defined as <50 nmol/L. The sequence was analyzed using the 25 alleles available on the server, with the results sorted by affinity before submission.

For CD8^+^ T cell epitope prediction, the NetMHCcons 1.1 Server (http://www.cbs.dtu.dk/services/NetMHCcons/, accessed on 8 April 2025) was employed. The FASTA file of the amino acid sequence was imported, setting the peptide length to between 8 and 11 mers and the IC50 threshold to <50 nmol/L. The analysis was performed using the server’s 25 alleles, with the results sorted by affinity prior to submission.

B cell epitope prediction was conducted using the IEDB online tool (http://tools.iedb.org/bcell/, accessed on 8 April 2025, Vesrion 2.0), wherein the amino acid sequence of LprO was inputted. The BepiPred Linear Epitope Prediction 2.0 tool was selected to identify B cell epitopes for the LprO protein [16].

### 2.2. Structural Prediction of LprO Protein

The AlphaFold 3 online platform (Version 3.0) was used to predict and analyze the three-dimensional structure of the LprO protein based on its amino acid sequence. The pLDDT score, ranging from 0 to 100, was employed to assess the reliability of the predicted structure, with higher scores indicating greater confidence [17]. The resultant LprO protein structure was annotated and visualized using PyMOL (Version 3.1.1).

### 2.3. Strains Cultivation and Growth Conditions

*Mycobacterium bovis* BCG (ATCC 927) and *Mycobacterium tuberculosis* H37Rv were cultured in Middlebrook 7H9 broth (Franklin Lakes, BD, USA), supplemented with 0.2% glycerol, 10% OADC (oleic acid, bovine serum albumin, dextrose, and catalase; Difco), and 0.5% Tween 80, or on 7H11 agar, containing 0.2% glycerol and 10% OADC, at 37 °C. *Escherichia coli* DH5α and BL21(DE3) were grown in Luria–Bertani medium (Sangon Biotech, Shanghai, China) or on agar for cloning and expression purposes.

### 2.4. Molecular Cloning

To construct pcDNA-*lprO*, primers were designed based on the genomes of M. tb. The *lprO* gene was amplified via PCR (Vazyme, Nanjing, China). During the construction process, the signal peptide sequence of the LprO protein was removed, a Kozak sequence was appended to the 5′ end of the *lprO* gene, and a Flag tag was incorporated at the 3′ end. The resulting pcDNA-*lprO* constructs were sequenced by Shanghai Qingke Biotechnology Co., Ltd., (Qingke, Shanghai, China) and analyzed using SnapGene software (Version 6.0.2) to verify the accuracy of the gene sequence.

### 2.5. Intracellular Expression Level Analysis

Following the manufacturer’s protocol for the Lipo8000™ (Beyotime, Shanghai, China) transfection reagent, DNA was transfected into HeLa cells. After 48 h, the cell culture supernatant was discarded, and the cells were washed with 1 mL of sterile PBS, which was subsequently removed. Each well was treated with 100 μL of RIPA buffer (Sangon Biotech, Shanghai, China) for cell lysis. Following thorough lysis, the lysate was collected and centrifuged at 12,000 rpm for 30 min at 4 °C. The resulting supernatant was mixed with 5× protein loading buffer and boiled for 15 min in a metal bath to ensure complete protein denaturation. A second centrifugation at 12,000 rpm for 10 min was performed before loading the total protein onto an SDS-PAGE gel, which was run at 120 V for 60 min. The proteins were transferred from the SDS-PAGE gel to a PVDF membrane using the eBlot™ L1 Rapid Wet Transfer System(GenScript, Nanjing, China) for Western blot analysis. The LprO protein was detected using an anti-Flag antibody, while GAPDH was identified using an anti-GAPDH antibody (Sangon Biotech, Shanghai, China). The PVDF membrane was incubated with the appropriate secondary antibodies and developed using a chemiluminescent substrate. Imaging was conducted using the LAS4000 imaging system.

### 2.6. Construction of Recombinant Strains and qRT-PCR Analysis

The vector construction strategy involved inserting the *lprO* gene, along with its 300 bp upstream sequence and a Flag tag, into the Hind III restriction site of the pNBV1 plasmid. The pNBV1-*lprO* construct was sequenced by Shanghai Qingke Biotechnology Co., Ltd. Upon sequence verification, the vector was electroporated into BCG Japan, with pNBV1 alone introduced as a control strain. Streptomycin-resistant colonies of BCG Japan::pNBV1 and BCG Japan::pNBV1-*lprO* were isolated. Following colony expansion, RNA was extracted using Trizol (Invitrogen, Carlsbad, CA, USA), and the total RNA was reverse transcribed into cDNA using the TAKARA reverse transcription kit. A quantitative real-time PCR (qRT-PCR) was then conducted, following the Qiagen qRT-PCR detection kit instructions to assess the transcriptional levels of *lprO*.

### 2.7. Detection of Secreted Proteins in BCG Culture Supernatant

Activated BCG Japan::pNBV1 and BCG Japan::pNBV1-*lprO* strains were inoculated in 100 mL of streptomycin-supplemented 7H9 medium and incubated at 37 °C until the optical density at 600 nm (OD_600_) reached 0.8–1.2. The cultures were centrifuged at 4000 rpm for 10 min to pellet the bacteria, which were then transferred into 50 mL centrifuge tubes. The pellets were resuspended in 50 mL of Sauton’s medium, centrifuged again at 4000 rpm for 10 min, and the supernatant discarded. The bacterial pellets were fully resuspended in fresh Sauton’s medium within sterile conical flasks, adjusting the OD_600_ to 0.6. The cultures were incubated at 37 °C in a shaking incubator until they reached the logarithmic growth phase (OD_600_ 1.0–1.2). The bacterial pellets were collected through centrifugation at 4000 rpm for 10 min, and the supernatant was carefully collected. This supernatant was filtered through a 0.22 μm membrane to eliminate any residual bacterial cells and concentrated 50-fold using a 3000 Da ultrafiltration tube. The concentrated supernatant was utilized to prepare protein samples for the Western blot analysis. GroEL2, an intracellularly expressed protein in *Mycobacterium* (1:500 antibody dilution), served as a negative control, while Ag85B, a known secreted protein (1:500 antibody dilution), functioned as a positive control.

### 2.8. Macrophage Infection and Cell Viability Assessment

Raw264.7 macrophages were seeded in a 96-well plate, at a density of 1 × 10⁴ cells per well, and cultured in 100 μL of 1640 cell culture medium for 20 h prior to infection. For each time point, a separate 96-well plate was utilized. The outermost wells contained the control cells (Raw264.7 cells + 1640 medium), while the wells with only 1640 medium served as blanks. The cells were infected at multiplicities of infection (MOIs) of 1 and 50. After 4 h of incubation, the culture supernatant was discarded, and the cells were washed twice with sterile PBS. Subsequently, 100 μL of 1640 medium containing 50 μg/mL of gentamicin was added, followed by a further two-hour incubation period. The medium was then discarded, the cells were washed twice with PBS, and fresh medium containing 100 units/mL of penicillin and 100 μg/mL of streptomycin was added for continued incubation. Cell viability was assessed at 4, 24, 48, 72, and 96 h post-infection. At each time point, the culture medium was removed, and 100 μL of 1640 medium was added to each well, followed by 10 μL of CCK-8 solution (Beyotime, Shanghai, China). The plate was gently shaken and incubated at 37 °C for 1–4 h until an orange color developed. Absorbance was measured at 450 nm to quantify cell viability.

### 2.9. Analysis of BCG Virulence in SCID Mice

Six-to-eight-week-old female SCID mice were obtained from Shanghai Slake Biological Technology Co., Ltd. (Slake, Shanghai, China) and maintained in a specific pathogen-free (SPF) grade animal facility. Following an acclimatization period of approximately one week, the mice were used for the experimental procedures. BCG Pasteur served as the positive control, while BCG Japan::pNBV1 functioned as the negative control. BCG Japan::pNBV1-*lprO* (with two biological replicates) was used as the experimental group, and sterile PBS acted as the blank control. Each mouse received a 100 μL tail vein injection of either bacterial suspension (containing an OD_600_ of 0.5) or sterile PBS, with 15 mice assigned per group. On day one and at week four post-injection, one mouse from each group, excluding the PBS control, was euthanized. The lungs and spleens were harvested and placed into grinding tubes containing 1 mL of sterile PBS, supplemented with 10% glycerol. The tissues were homogenized at 5000 rpm for 20 s, repeated three times, using a tissue homogenizer. The homogenate was serially diluted and plated, followed by incubation at 37 °C for four weeks to determine the actual inoculation dose. The mice were weighed weekly to monitor body weight fluctuations. Survival was recorded daily, and survival curves were plotted to evaluate the mortality rates across all the groups.

### 2.10. Immunogenicity Assessment

For the immunogenicity analysis, BCG-immunized C57BL/6J mice received subcutaneous injections of ~10^6^ CFU of either BCG Japan::pNBV1 or BCG Japan::pNBV1-*lprO*. DNA-immunized C57BL/6J mice were intramuscularly injected with 100 μg/100 μL of either pcDNA or pcDNA-*lprO*, administered in three immunizations at two-week intervals. The blank control group received 100 μL of sterile PBS. Three weeks after the final DNA immunization, spleen lymphocytes were isolated from all the mice. The cells were plated at a density of 2 × 10^6^ cells/well in 48-well plates, with each sample divided into two groups: one stimulated with PPD and the other left unstimulated. After 30 h of incubation, 4 μL of Monensin Solution (diluted 10-fold) was added for an additional 6 h of stimulation. For the positive control group, 50 ng/mL of PMA and 500 ng/mL of ionomycin were added, alongside the Monensin Solution for 5 h of stimulation. Post-stimulation, the cells were collected and subjected to flow cytometry analysis. The cells were transferred into tubes and centrifuged at 4 °C, 1700 rpm for 5 min. The pellet was washed with 1 mL of Staining Buffer, centrifuged again, and the supernatant discarded. The cells were resuspended in 100 μL of Staining Buffer, followed by the addition of 4 μL of TruStain FcX™ (anti-mouse CD16/32, Biolegend, San Diego, CA, USA) and incubated at 4 °C in the dark for 15 min. Next, 1 μL of Zombie Aqua™ Fixable Viability Kit (Biolegend)dye was added, and the cells were incubated at 4 °C in the dark for another 15 min. After washing with Staining Buffer and centrifugation, the cells were resuspended in 100 μL of Staining Buffer and stained sequentially with APC/Fire™ 750 anti-mouse CD3 (Biolegend), Alexa Fluor^®^ 700 anti-mouse CD4 (Biolegend), PerCP/Cyanine5.5 anti-mouse CD8a (Biolegend), PE anti-mouse/human CD44 (Biolegend), and FITC anti-mouse CD62L (Biolegend), following the manufacturer’s instructions.

Incubate the cells at 4 °C in the dark for 20 min. Wash the cells with 1 mL of Staining Buffer, followed by centrifugation at 4 °C, 1700 rpm for 5 min, and discard the supernatant. Add 500 μL of fixation buffer (Biolegend), gently mix through pipetting, and incubate again at 4 °C in the dark for 20 min. Centrifuge at 4 °C, 1700 rpm for 5 min, discard the supernatant, and add 500 μL of 1× perm/wash buffer. Gently mix through pipetting, centrifuge at 4 °C, 1700 rpm for 5 min, and discard the supernatant. Repeat this washing step twice. Resuspend the cells in 100 μL of Staining Buffer and sequentially add APC anti-mouse TNF-α (Biolegend), PE/Dazzle™ 594 anti-mouse IFN-γ (Biolegend), and Brilliant Violet 421™ anti-mouse IL-2 (Biolegend), following the manufacturer’s instructions. Incubate at 4 °C in the dark for 20 min. Wash the cells with 1 mL of 1× perm/wash buffer (Biolegend), centrifuge at 4 °C, 1700 rpm for 5 min, and discard the supernatant. Perform an additional wash using 1 mL of Staining Buffer, centrifuge in identical conditions, and discard the supernatant. Finally, resuspend the cells in 300 μL of Staining Buffer and proceed with flow cytometry analysis.

### 2.11. Animal Protection Studies

To evaluate the anti-tuberculosis protective efficacy of the vaccine using a zebrafish (*Danio rerio*) *M. marinum* infection model, AB strain zebrafish aged 3–4 months were sourced from Nanjing Yishuli Hua and acclimated for one week before experimentation. The zebrafish were anesthetized with 500 mg/L of tricaine, which was injected into the dorsal muscle using a microinjection syringe. Each fish received 2 μL of solution, containing a total DNA dose of 6 μg. The blank control group was injected with an equivalent volume of sterile PBS. Immediately following injection, electroporation was performed using six 50 V pulses, each lasting 5 ms. The pcDNA-immunized group served as the negative control, while the experimental groups were immunized with pcDNA-*lprO*. Immunizations were administered twice at two-week intervals. Two weeks after the final immunization, the zebrafish were infected with 200 CFU of *M. marinum* 535 per fish via intraperitoneal injection, and survival was monitored post-infection.

To further assess the vaccine’s protective efficacy, a mouse BCG Pasteur infection model was employed. Four-week-old female BALB/c mice were purchased from Shanghai Slake Biological Technology Co., Ltd. and housed in an SPF animal facility. After one week of acclimatization, the mice were used for experimentation. Each mouse received a subcutaneous injection of ~10^6^ CFU of either BCG Japan::pNBV1 or BCG Japan::pNBV1-*lprO*. The blank control group was injected with 100 μL of sterile PBS. Eight weeks post-injection, the mice were challenged via tail vein injection with ~10^7^ CFU of BCG Pasteur. Three weeks following the challenge, lung and spleen tissues were harvested from the mice to determine the bacterial load.

To assess the protective efficacy of the vaccine, a mouse M. tb infection model was employed. Four-week-old female BALB/c or C57BL/6J mice were procured from Jiangsu Jicui Pharmaceutical Biotechnology Co., Ltd. (GemPharmatech, Nanjing, China) and housed in SPF facilities for one week prior to experimentation. The mice in the BCG immunization groups received subcutaneous injections of ~10⁶ CFU of either BCG Japan::pNBV1 or BCG Japan::pNBV1-*lprO*. The mice in the DNA immunization groups were intramuscularly injected with 100 μg/100 μL of either pcDNA or pcDNA-*lprO*, with three immunizations administered at two-week intervals. The blank control group was injected with 100 μL of sterile PBS. Three weeks following the final DNA immunization, all the mice were infected with approximately 150 CFU of M. tb H37Rv via aerosol exposure. Four weeks post-infection, lung and spleen tissues were harvested to evaluate the bacterial load.

### 2.12. Histopathological Analysis

The zebrafish were anesthetized with tricaine and, subsequently, euthanized. Following euthanasia, the specimens were rinsed once with sterile PBS and fixed in 4% paraformaldehyde for one week. They were then rinsed overnight with running water. Decalcification was carried out by immersing the zebrafish in a 10% EDTA solution for one week. The samples were then dehydrated using a graded ethanol series and cleared with xylene. Tissue infiltration was performed using paraffin. The paraffin was filtered to remove impurities, and the zebrafish were immersed in paraffin at 60 °C for 2 h, followed by being transferred to fresh paraffin for overnight incubation to eliminate residual xylene. Embedding was completed by placing the zebrafish in molds containing molten paraffin, ensuring the removal of air bubbles. After natural solidification, the blocks were cooled at −30 °C for 3–5 min, demolded, and sent to Shanghai Ruibaohe Biotechnology Co., Ltd. for sectioning. The returned sections underwent deparaffinization. The sections were immersed in hematoxylin for 10 min, rinsed with running water until clear, and differentiated in 1% hydrochloric acid for 2 s, followed by further rinsing.

They were then immersed in eosin for 5 s and rinsed thoroughly with running water. After drying, the sections were cleared in Van-Clear I (Servicebio, Wuhan, China) for 10 min and Van-Clear II for 5 min. Once dried, the sections were mounted with coverslips and examined microscopically. Sections containing intact zebrafish viscera were selected for acid-fast staining. After deparaffinization, sections were immersed in carbol fuchsin solution for 20 min, rinsed with tap water, and differentiated in acid alcohol for 2–5 s. Decolorization was repeated until red, rod-shaped bacteria were visible under the microscope. Sections were then counterstained with methylene blue for 3–5 s, rinsed, dried, mounted with coverslips, and examined microscopically.

## 3. Results

### 3.1. Prediction of T and B Cell Epitopes in LprO Protein

T cell epitope prediction for the LprO protein was conducted using the NetMHC II 2.3 Server (Version 2.3)and NetMHCcons 1.1 Server (Version 1.1) to identify peptides capable of binding to MHC II and MHC I molecules, corresponding to CD4^+^ and CD8^+^ T cell epitopes, respectively. As a reference, the Ag85A antigen (338 amino acids), recognized for its strong immunogenicity and comparable size to LprO (369 amino acids), was analyzed concurrently. In the case of LprO, 692 MHC II-binding sites, including 134 strong binding sites, and 69 MHC I binding sites, with 16 strong binding sites, were identified (Table S1). In contrast, Ag85A exhibited 546 MHC II binding sites and 71 MHC I binding sites, indicating that the epitope count for LprO is comparable to that of Ag85A (Table S1).

To further assess the immunogenic potential of LprO, B cell epitope prediction was performed using the IEDB database. This analysis identified B cell epitopes covering 177 amino acids, representing 47.96% of the total LprO sequence, suggesting considerable potential to elicit humoral immune responses (Figure S1). An integrated analysis of the predicted CD4^+^ T, CD8^+^ T, and B cell epitopes revealed eight peptide sequences, ranging from 6 to 12 amino acids in length, that were common across all three immune response pathways (Table S2). These findings highlight LprO’s significant immunogenic potential, comparable to Ag85A, in stimulating both cellular and humoral immunity.

The structural prediction of the LprO protein was further refined using AlphaFold 3 (Version 3.0). The protein consists of 369 amino acids, with the N-terminal 34 residues extending beyond the core structure and displaying low pLDDT scores (50–70), indicating an unstable region. According to UniProt (https://www.uniprot.org/uniprotkb/O07423/entry, accessed on 8 April 2025, ID: O07423_MYCTU), this segment is predicted to function as a signal peptide. The core structure of LprO, following the signal peptide, is well-defined and comprises nine alpha-helices and sixteen beta-strands, forming a compact and stable architecture (Figure S2).

**Figure 1:**
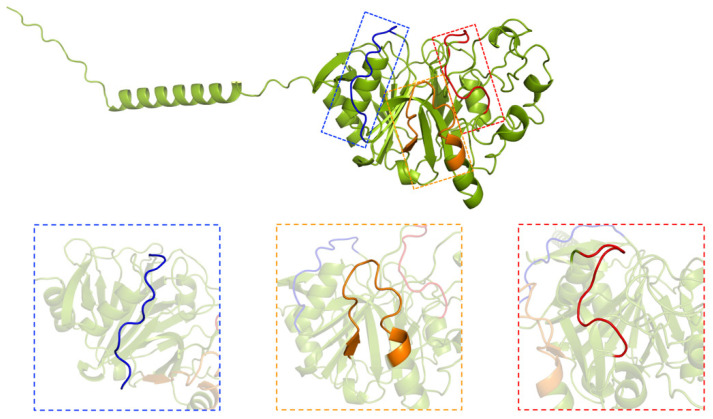
The 3D structure of the LprO protein predicted by AlphaFold 3.

Three shared T cell and B cell epitopes, each exceeding ten amino acids in length, were mapped onto the predicted structure: the red segment (AGKQGLSGGNE), the orange segment (YVLRPKSRQDYDLATP), and the blue segment (GNTGQLHDGGPS). These epitopes are predicted to adopt flexible loop conformations and are situated on the protein surface, enhancing their accessibility for interactions with host immune molecules (Figure 1). Such surface exposure may facilitate immune recognition and contribute to the protein’s role in modulating host immune responses.

### 3.2. The pcDNA-lprO Enhances the Level of M. tb-Specific IFN-γ-Secreting Lymphocytes in Mouse Spleen

**Figure 2:**
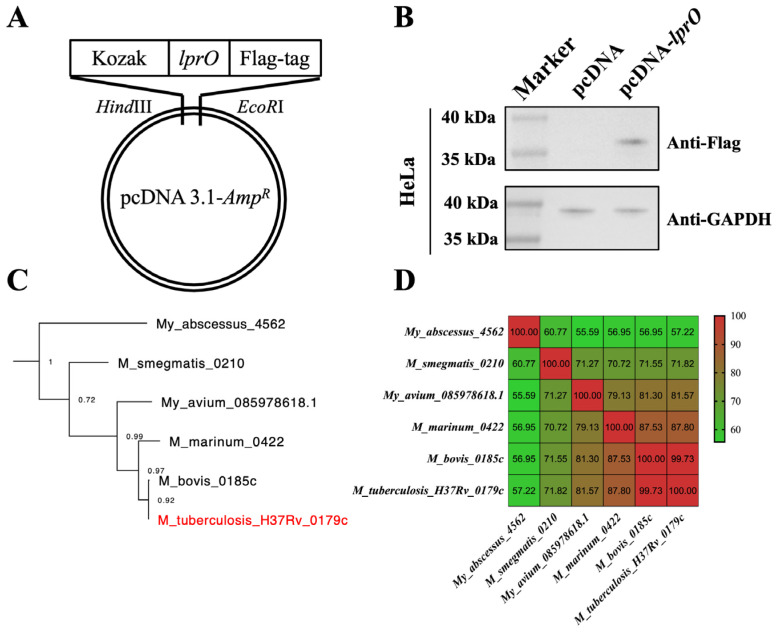
Homology comparison of mycobacterial LprO protein and construction of pcDNA-*lprO*. (**A**) Schematic diagram of pcDNA-*lprO* construction. (**B**) Western blot analysis of intracellular expression levels of pcDNA-*lprO* in HeLa cell lines, 24 h post-transfection. (**C**) Phylogenetic tree of mycobacterial LprO protein. The LprO protein derived from M. tb is indicated in red. (**D**) Heatmap comparing the sequence similarity of LprO protein genes from various *Mycobacterium* species.

To further assess the potential of LprO as a TB vaccine candidate, a DNA vaccine was developed using the LprO protein as the antigen and the pcDNA 3.1 plasmid as the vector. Constructs of pcDNA-*lprO* were generated from M. tb and designated pcDNA-*lprO* (Figure 2A). The recombinant vaccines were then transfected into HeLa cells to evaluate their expression in eukaryotic systems. The results confirmed the successful expression of pcDNA-*lprO* in eukaryotic cell lines (Figure 2B).

**Figure 3:**
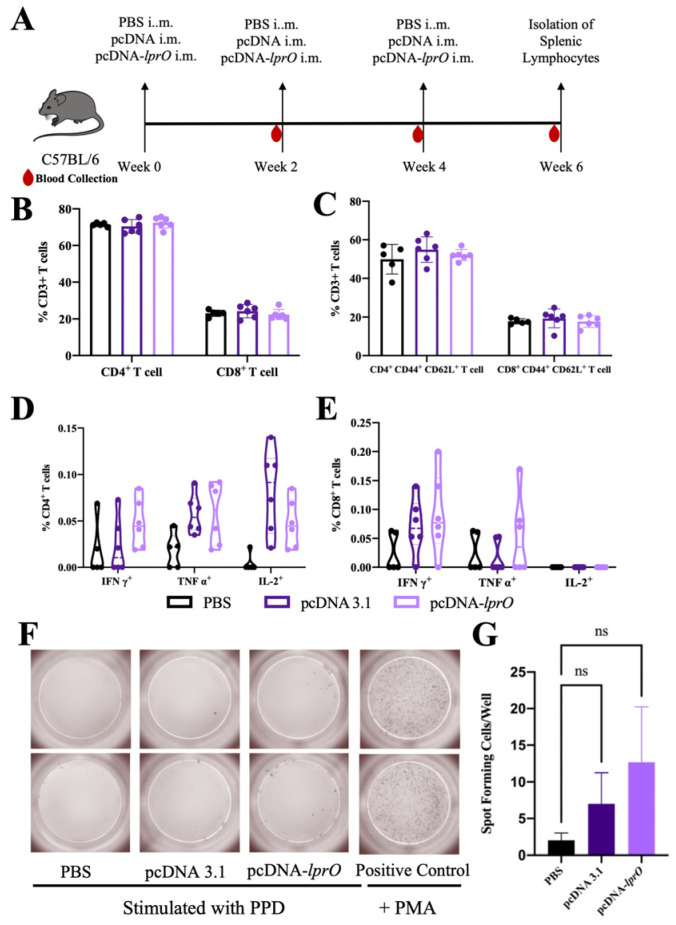
Antigen-specific immune response of splenic lymphocytes in mice immunized with pcDNA-*lprO*. (**A**) Schematic diagram of the C57BL/6J mice immunization process with pcDNA-*lprO*. Mice were immunized intramuscularly with PBS, pcDNA, or pcDNA-*lprO*, receiving three immunizations administered every two weeks (n = 5/6 per group). Two weeks after the final immunization, spleen lymphocytes from some mice were stimulated with LprO protein for 30 h, followed by surface and intracellular cytokine staining and flow cytometry analysis to determine cell proportions. Red droplets indicate blood collection from mice one day prior to immunization or before euthanasia. (**B**) Proportion of CD4^+^ and CD8^+^ T cells. (**C**) Proportion of memory T cells. (**D**,**E**) Proportion of different subpopulations of CD4^+^ and CD8^+^ T cells. (**F**,**G**) Proportion and quantification of INF-γ^+^ secreting lymphocytes detected using an Elispot assay. Data are presented as mean ± SD. Statistical analysis was performed using a two-way ANOVA, with comparisons made against the PBS group; NS indicates not significant (*p* > 0.05).

To investigate immune correlates of protection in pcDNA-*lprO*-immunized mice, the antigen-specific adaptive T cell response was analyzed in the spleens of PBS, pcDNA, and pcDNA-*lprO*-vaccinated C57BL/6J mice two weeks post-immunization (Figure 3A). There were no significant differences in the proportions of CD4+, CD8+, and memory T cells (CD3^+^ CD44^+^ CD62L^+^) across the groups following LprO protein stimulation (Figure 3B,C). Similarly, the proportions of antigen-specific IFN-γ+, TNF-α+, or IL-2+ T cells did not differ notably compared to the PBS and pcDNA groups (Figure 3D,E). However, the pcDNA-*lprO* vaccine did increase the proportion of antigen-specific CD8^+^ TNF-α^+^ T cells (Figure 3E). Subsequently, the levels of IFN-γ secreted by splenic lymphocytes from pcDNA-*lprO*-immunized mice were measured following PPD stimulation. Compared to the control groups, splenocytes from pcDNA-*lprO*-vaccinated mice exhibited a higher proportion of tuberculosis-specific IFN-γ-secreting lymphocytes, ranging from 0.5% to 3% (Figure 3F,G).

These findings indicate that LprO, as a DNA vaccine, can elicit a detectable immune response and enhance the CD8^+^ T cell response to a measurable extent, although the differences in regard to the control group were not statistically significant.

### 3.3. The pcDNA-lprO Vaccine Protects Zebrafish Against M. marinum Infection

**Figure 4:**
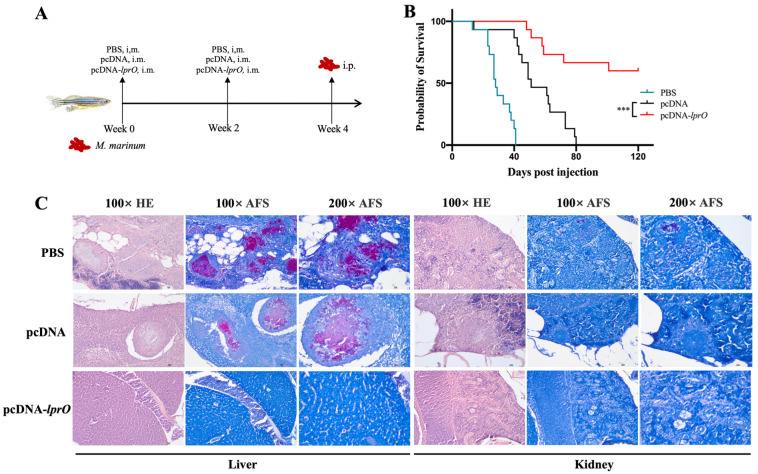
The pcDNA-*lprO* vaccine protects zebrafish against *M. marinum* infection. (**A**) Schematic representation of vaccine immunization and *M. marinum* infection. Each fish was assigned to one of the following groups: PBS, pcDNA, or pcDNA-*lprO*-. Immunization was performed via intramuscular injection (i.m.), with two immunizations administrated at a two-week interval, and each fish received 6 µg of DNA. Two weeks after the final immunization, each fish was intraperitoneally (i.p.) infected with 200 CFU of *M. marinum*. (**B**) Survival curves of zebrafish from different immunization groups following *M. marinum* infection (n = 15 per group). Statistical analysis was performed using the log rank (Mantel–Cox) test; NS indicates not significant; *** *p* < 0.001. (**C**) Hematoxylin–eosin (HE) staining and acid-fast staining (AFS) of zebrafish pathological sections following *M. marinum* infection.

The protective efficacy of the pcDNA-*lprO* vaccine against tuberculosis was examined using a *Mycobacterium marinum* (*M. marinum*) infection model in zebrafish (Figure 4A). The survival curves revealed that the zebrafish in the PBS control group had a median survival time of 28 days, whereas the pcDNA group survived for a median of 51 days. The pcDNA-*lprO* vaccine significantly improved the survival rate of infected zebrafish (Figure 4B).

The pathological analyses revealed dense granulomatous tissue in the liver and kidneys of zebrafish from the PBS and pcDNA control groups, with necrotic centers and widespread dissemination of *M. marinum* 535. In contrast, the zebrafish in the pcDNA-*lprO* group exhibited normal liver and kidney structures, with no observable granulomatous lesions (Figure 4C). These results suggest that the pcDNA-*lprO* vaccine offers enhanced protection against tuberculosis, as evidenced by the increased survival rates and reduced bacterial burden in infected zebrafish.

### 3.4. Recombinant BCG Japan::pNBV1-lprO Secretes LprO Protein

**Figure 5:**
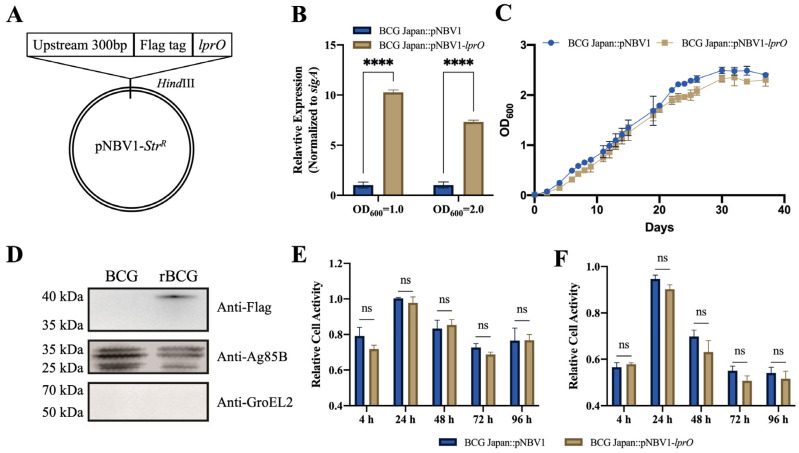
BCG Japan::pNBV1-*lprO* was successfully constructed, capable of secreting LprO-Flag protein, without affecting the in vitro growth or virulence. (**A**) Schematic diagram of the construction of the pNBV1-*lprO* plasmid. (**B**) The qRT-PCR validation of the transcriptional levels of LprO protein. Data are presented as mean ± SD. Statistical analysis was performed using a two-way ANOVA; **** *p* < 0.0001. (**C**) In vitro growth curves of BCG Japan::pNVB1 and BCG Japan::pNVB1-*lprO*. (**D**) Western blot analysis of LprO-Flag protein secretion in BCG (BCG Japan::pNVB1) and rBCG (BCG Japan::pNVB1-*lprO*). (**E**,**F**) Cell viability levels after infection of Raw264.7 cells with MOI = 1 and MOI = 50, respectively. Data are presented as mean ± SD. Statistical analysis was performed using a two-way ANOVA; NS indicates not significant.

The BCG vaccine remains the only approved vaccine for TB, while LprO, utilized as a DNA vaccine, has demonstrated promising protective effects against TB. To further assess the potential of a recombinant BCG as a TB vaccine, a BCG Japan strain overexpressing LprO was engineered (Figure 5A). Cultures of BCG Japan::pNBV1 and BCG Japan::pNBV1-*lprO* were harvested during both logarithmic and stationary growth phases to evaluate *lprO* transcription levels. The expression of *lprO* in BCG Japan::pNBV1-*lprO* was markedly higher than in BCG Japan::pNBV1 across both growth phases (Figure 5B). Notably, *lprO* transcript levels during the logarithmic phase were approximately double those of the stationary phase, leading to the selection of the logarithmic phase strain for subsequent experiments (Figure 5B). Additionally, the LprO protein contains a signal peptide, enabling its secretion. Supernatants from BCG Japan::pNBV1-*lprO* cultures were collected, and the Western blot analyses confirmed that the LprO protein was secreted into the culture medium (Figure 5D). Importantly, LprO overexpression did not affect the in vitro growth rate of BCG Japan (Figure 5C).

The virulence of the recombinant BCG strain was then assessed at the cellular level. Raw264.7 macrophages were infected with BCG Japan::pNBV1-*lprO* at multiplicities of infection (MOIs) of 1 (Figure 5E) and 50 (Figure 5F). Cell viability remained comparable to that observed in regard to the control strain, BCG Japan::pNBV1, indicating similar virulence in macrophages regardless of the infection dose.

### 3.5. Recombinant BCG Japan-pNBV1-lprO Vaccine Exhibits an Excellent Safety Profile

**Figure 6:**
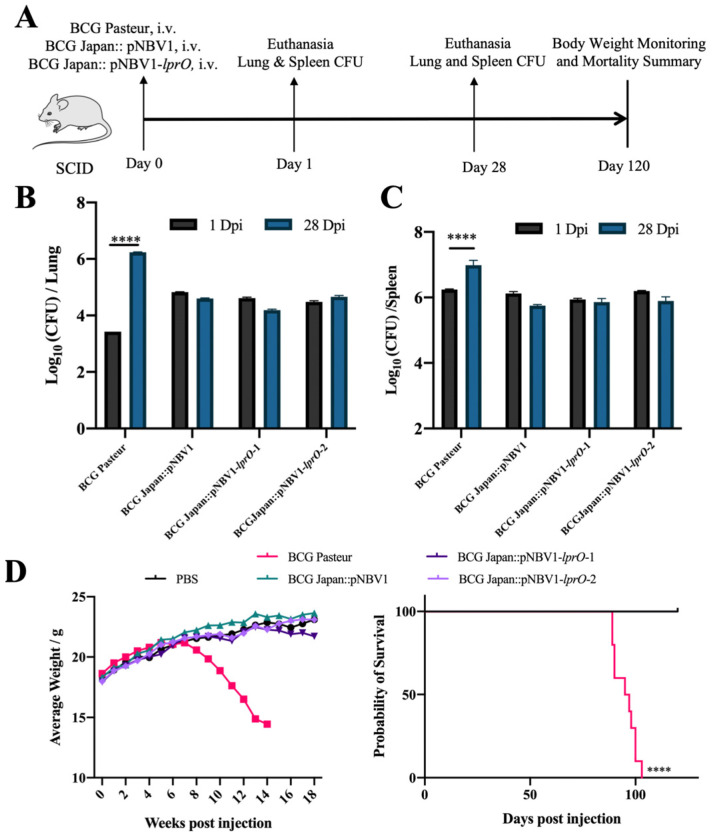
The BCG Japan::pNBV1-*lprO* vaccine demonstrated good safety. (**A**) Timeline for safety assessment experiments of BCG Japan::pNBV1 and BCG Japan::pNBV1-*lprO*. BCG Pasteur was used as a positive control. Mice were infected with ~10^6^ CFU of bacteria. On day 1 post-infection, one mouse per group was selected, and on day 28, four mice per group were selected to assess the bacterial load in the lungs and spleens. Mouse weights were recorded weekly, and mortality was monitored over a total experimental period of 120 days. (**B**,**C**) Bacterial load in the lungs and spleens of SCID mice on day 1 and day 28 post-infection with BCG Pasteur, BCG Japan::pNBV1, BCG Japan::pNBV1-*lprO*-1, and BCG Japan::pNBV1-*lprO*-2. Data are presented as mean ± SD. Statistical analysis was performed using a two-way ANOVA; NS indicates not significant; ****: *p* < 0.0001. (**D**) Weight changes and survival curve analysis in SCID mice infected with BCG Pasteur, BCG Japan::pNBV1, BCG Japan::pNBV1-*lprO*-1, and BCG Japan::pNBV1-*lprO*-2. Statistical analysis was performed using the log rank (Mantel–Cox) test; *ns* indicates not significant; **** *p* < 0.0001.

The safety profile of the recombinant BCG was evaluated using SCID mice (Figure 6A). On the first day post-injection, no significant differences in the bacterial loads were detected in the spleens and lungs across all the groups. By the fourth week, the BCG Pasteur group exhibited a notable increase in the bacterial loads in both organs. In contrast, the bacterial loads in the lungs and spleens of mice in the BCG Japan::pNBV1, BCG Japan::pNBV1-*lprO*-1, and BCG Japan::pNBV1-*lprO*-2 groups remained consistent (Figure 6B,C). The proliferation capacity of the BCG Japan::pNBV1-*lprO* strain was comparable to that of BCG Japan::pNBV1.

The body weight of the mice was monitored weekly post-injection. In the later stages, the mice in the highly virulent BCG Pasteur group exhibited yellowed fur, reduced appetite, decreased activity, and significant weight loss. Conversely, mice in the low-virulence BCG Japan::pNBV1 and BCG Japan::pNBV1-*lprO* groups showed only mild fur dullness, with stable appetite and activity levels, similar to the PBS group, and their body weight gradually increased (Figure 6D). Survival was tracked across ten mice per group. All the mice in the BCG Pasteur group died within 105 days post-infection, whereas no deaths occurred in the other groups over the 120-day study period (Figure 6D). The BCG Japan::pNBV1-*lprO* strain demonstrated virulence levels similar to BCG Japan::pNBV1, both significantly lower than the BCG Pasteur group, indicating a high safety margin in SCID mice. Overall, the strains exhibiting enhanced in vivo proliferation generally display higher virulence; however, the proliferation of BCG Japan::pNBV1-*lprO* in SCID mice aligned with its lower virulence profile.

### 3.6. Immunization with Recombinant BCG Japan-pNBV1-lprO Enhances M. tb-Specific Th1 Immune Response in Mice

**Figure 7:**
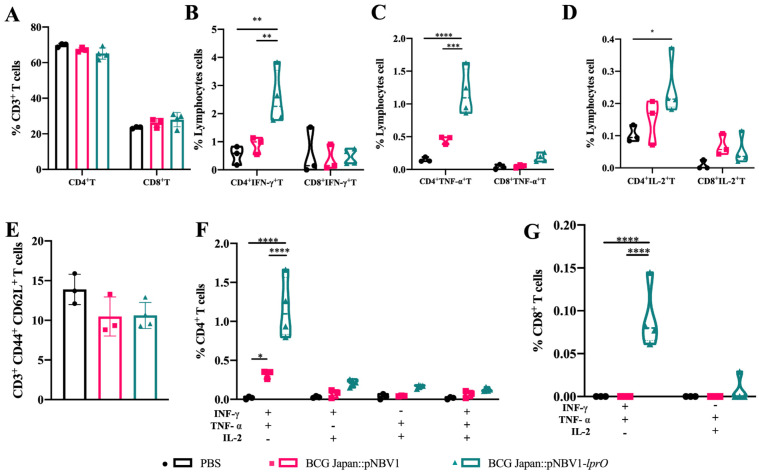
Immunization with recombinant BCG Japan-pNBV1-*lprO* enhances Tuberculosis-Specific Th1 immune response in mice. C57BL/6J mice were immunized subcutaneously with PBS, BCG Japan::pNBV1, or BCG Japan::pNBV*1-lprO*, receiving ~10^6^ CFU per mouse. Eight weeks post-immunization, spleen lymphocytes were isolated from the C57BL/6J mice. After 30 h of PPD stimulation, cells were stained for surface and intracellular cytokines and analyzed using flow cytometry to determine cell proportions (n = 4 per group). (**A**) Proportion of CD4^+^ and CD8^+^ T cells. (**B**–**D**,**F**,**G**) Proportion of different subpopulations of CD4^+^ and CD8^+^ T cells. (**E**) Proportion of memory T cells. Data are presented as mean ± SD. Statistical analysis was performed using a two-way ANOVA; NS indicates not significant; *: *p*< 0.05; **: *p* < 0.01; ***: *p* < 0.001; ****: *p* < 0.0001.

The splenic T cell responses were analyzed in C57BL/6J mice following subcutaneous immunization with BCG Japan::pNBV1-*lprO* or BCG Japan::pNBV1. Eight weeks post-immunization, splenic lymphocytes were co-cultured with PPD, followed by surface and intracellular cytokine staining and flow cytometry analysis. After PPD stimulation, no significant differences were observed between the groups in terms of the numbers of CD4^+^, CD8^+^ T cells, or memory T cells (CD3^+^ CD44^+^ CD62L^+^). Overexpression of *lprO* did not affect T cell subpopulation distribution (Figure 7A,E). The proportions of CD4^+^ and CD8^+^ T cells expressing intracellular cytokines IFN-γ, TNF-α, and IL-2 were measured. The percentage of single cytokine-positive CD4^+^ T cells exceeded that of CD8^+^ T cells. No significant differences were found between the groups for CD8^+^ IFN-γ^+^ or CD8^+^ IL-2^+^ T cells (Figure 7B–D). However, the proportion of CD8^+^ TNF-α^+^ T cells was elevated in the BCG Japan::pNBV1-*lprO*-immunized group compared to the PBS and BCG Japan::pNBV1 groups (Figure 7C). Furthermore, the percentages of CD4^+^ IFN-γ^+^, CD4^+^ TNF-α^+^, and CD4^+^ IL-2^+^ T cells were significantly higher in the BCG Japan::pNBV1-*lprO*-immunized group than in the BCG Japan::pNBV1 group (Figure 7B–D). These findings suggest that immunization with BCG Japan::pNBV1-*lprO* induces a stronger CD4^+^ T cell response, with CD4^+^ IFN-γ^+^ T cells being the most dominant and playing a central role in the immune response.

T cells co-expressing two or three cytokines are classified as multifunctional T cells. The proportions of double-positive and triple-positive CD8^+^ T cells remained low, with minimal detection of CD8^+^ TNF-α^+^ IL-2^+^ and CD8^+^ IFN-γ^+^ IL-2^+^ T cells across all the groups. Detectable levels of CD8^+^ IFN-γ^+^ TNF-α^+^ and CD8^+^ IFN-γ^+^ TNF-α^+^ IL-2^+^ T cells were found exclusively in the BCG Japan::pNBV1-*lprO* group, indicating a moderate enhancement in CD8^+^ T cell responses (Figure 7G). In contrast, the proportion of double- and triple-positive CD4^+^ T cells was significantly increased, with CD4^+^ IFN-γ^+^ TNF-α^+^ T cells notably higher in the BCG Japan::pNBV1-*lprO* group. Additionally, increases were observed in CD4^+^ TNF-α^+^ IL-2^+^, CD4^+^ IFN-γ^+^ IL-2^+^, and CD8^+^ IFN-γ^+^ TNF-α^+^ IL-2^+^ T cells (Figure 7F). These results indicate that BCG Japan::pNBV1-*lprO* enhances host M. tb-specific Th1 immune responses and has potential applications in regard to anti-tuberculosis protection.

### 3.7. Immunization with Recombinant BCG-lprO and pcDNA-lprO Reduces M. tb Load in the Lungs of Mice

**Figure 8:**
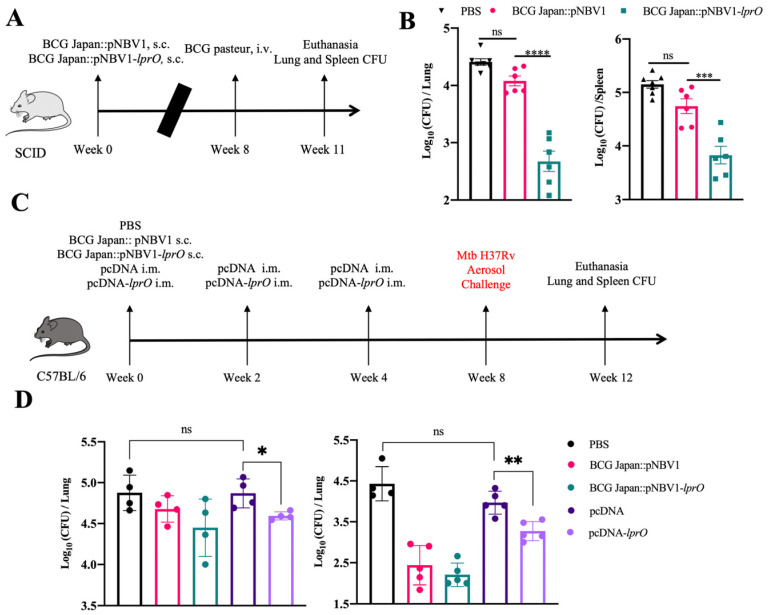
Immunization with recombinant BCG-*lprO* and pcDNA-*lprO* reduces M. tb load in the lungs of mice. (**A**) Timeline for evaluating the anti-tuberculosis protective efficacy of recombinant BCG in a mouse-BCG Pasteur infection model. Mice were immunized subcutaneously with either BCG Japan::pNBV1 or BCG Japan::pNBV1-*lprO*, receiving ~10^6^ CFU per mouse. Eight weeks post-immunization, mice were infected with ~10^7^ CFU of BCG Pasteur via a tail vein injection. Bacterial load in the lungs and spleens were assessed three weeks post-infection. (**B**) Bacterial load in the lungs and spleens of mice in the mouse BCG Pasteur infection model (n = 6 per group). Data are presented as mean ± SD. Statistical analysis was performed using an ordinary one-way ANOVA with multiple comparisons; ****: *p* < 0.0001, ***: *p* < 0.002. (**C**) Timeline for assessing the anti-tuberculosis protective efficacy of recombinant BCG and pcDNA-*lprO* in a mouse M. tb infection model. Mice were subcutaneously immunized with either BCG Japan::pNBV1 or BCG Japan::pNBV1-*lprO* (~10^6^ CFU). Mice in the DNA vaccine group were immunized intramuscularly with either pcDNA or pcDNA-*lprO*-M. tb, with three immunizations administered every two weeks. Four weeks after the final DNA vaccine immunization, mice were infected with M. tb via aerosol exposure. Bacterial loads and pathological analysis of the lungs were performed four weeks post-infection. Two rounds of experiments were conducted. (**D**) Bacterial load in the lungs of mice in the mouse M. tb infection model. The left graph displays the results from the first round of the experiments (n = 4 per group), where the average bacterial load in the lungs on day 1 post-infection was 128 CFU and the right panel shows the results from the second round of experiments (n = 5 per group), where the average bacterial load in the lungs on day 1 post-infection was 358 CFU. The data are presented as mean ± SD. Statistical analysis was performed using an unpaired *t*-test; NS indicates not significant; *: *p* = 0.0240; **: *p* = 0.0028.

To evaluate the protective efficacy of BCG Japan::pNBV1-*lprO* against tuberculosis, a high-dose infection model using a virulent BCG strain was employed in Balb/c mice. The mice were subcutaneously immunized with 1 × 10⁶ CFU, followed by a challenge of 1 × 10⁷ CFU of BCG Pasteur administered via a tail vein injection eight weeks later. Three weeks post-challenge, the bacterial loads in the lungs and spleen were quantified (Figure 8A). In the initial experiment, the mice immunized with BCG Japan::pNBV1-*lprO* exhibited a reduction in the lung bacterial load of 1.41 log_10_ and a spleen reduction of 0.93 log_10_ compared to the BCG Japan::pNBV1 control group (Figure 8B). In a subsequent experiment, the lung and spleen bacterial loads were reduced by 0.77 log_10_ and 0.59 log_10_, respectively (Figure S4). The data from both experiments confirm that BCG Japan::pNBV1-*lprO* effectively decreases the bacterial loads in both the lungs and spleen of mice.

To further assess the anti-tuberculosis efficacy of LprO as an overexpressed recombinant BCG antigen and as a single antigen in a DNA vaccine, the mice were subcutaneously immunized with either BCG Japan::pNBV1 or BCG Japan::pNBV1-*lprO* (~10⁶ CFU). For the DNA vaccine groups, the mice received intramuscular injections of either pcDNA or pcDNA-*lprO*, with three immunizations administered at two-week intervals. Four weeks after the final DNA vaccination, the mice were infected with M. tb via aerosol, and the lung bacterial loads were assessed four weeks post-infection (Figure 8C). This study was replicated across two independent trials. In the first trial, the BCG Japan::pNBV1-*lprO* group demonstrated a 0.24 log_10_ reduction in the lung bacterial load compared to the BCG Japan::pNBV1 group. Among the DNA vaccine groups, pcDNA-*lprO* yielded a 0.28 log_10_ reduction relative to pcDNA (Figure 8D). In the second trial, the BCG Japan::pNBV1-*lprO* group again showed a 0.24 log_10_ reduction, while pcDNA-*lprO*-M. tb achieved a 0.69 log_10_ reduction compared to pcDNA (Figure 8D). These findings from two independent trials highlight LprO’s potential as an anti-tuberculosis vaccine antigen, with the DNA vaccine format showing particularly promising protective efficacy.

## 4. Discussion

TB, caused by M. tb infection, is a chronic respiratory disease that has persisted for over 2000 years. Over the past two centuries, TB has been responsible for approximately one billion deaths and continues to pose a significant global health threat [18]. The BCG vaccine, first introduced for human TB prevention in 1921, remains the only licensed vaccine against TB [19]. As one of the longest used and most widely administered vaccines globally, BCG has demonstrated a robust safety profile. However, its efficacy as an anti-tuberculosis vaccine remains limited, offering only partial protection against pulmonary TB in children and inconsistent protection in adults (ranging from 0% to 80%), with its effectiveness diminishing with age [20].

Various strategies are being explored worldwide to develop new vaccines against tuberculosis: (1) the construction of recombinant BCG (rBCG) vaccines, (2) the development of subunit vaccines, and (3) the creation of vaccines based on alternative bacterial strains [21]. In this study, the potential of LprO as an antigen was investigated through the construction of recombinant BCG and DNA subunit vaccines. The *lprO* gene was overexpressed in BCG Japan to produce a live recombinant BCG vaccine (designated BCG Japan::pNBV1-*lprO*). Additionally, a DNA subunit vaccine encoding *lprO* was constructed using the pcDNA 3.1 vector (designated pcDNA-*lprO*). Using a *M. marinum*-infected zebrafish model, immunization with pcDNA-*lprO* significantly extended the survival of infected zebrafish. Furthermore, in a M. tb-infected mouse model, both LprO-based vaccines effectively reduced the lung bacterial burdens compared to their respective controls, although the differences compared to the control were not statistically significant for mice immunized with the recombinant BCG vaccine. Notably, pcDNA-*lprO*, as a DNA vaccine encoding a single antigen, achieved a substantial reduction in the lung bacterial load (by 0.28–0.69 log_10_ compared to the pcDNA group), highlighting the promising protective potential of LprO against tuberculosis.

The immunogenicity analyses of the two vaccine forms revealed that the recombinant BCG vaccine elicited a more robust CD4^+^ Th1 response, characterized by elevated levels of IFN-γ, TNF-α, and IL-2, as well as an increase in dual and triple cytokine-positive cells, alongside a modest CD8^+^ IFN-γ^+^ TNF-α^+^ T and CD8^+^ IFN-γ^+^ TNF-α^+^ IL-2^+^ T cell response. In contrast, the pcDNA-*lprO* vaccine predominantly induced a stronger antigen-specific CD8^+^ TNF-α^+^ T cell response. In the case of the recombinant BCG vaccine, overexpression of *lprO* resulted in alterations within the BCG strain itself, leading to changes in the expression of the genes beyond *lprO*, potentially contributing to immune responses distinct from the parental strain. This phenomenon is consistent with observations in terms of different BCG strains, which, due to varying deletion clusters, exhibit divergent immune profiles [22]. Sang et al. reported that overexpression of *phoP-phoR* in recombinant BCG Japan enhanced its protective capacity against tuberculosis. Transcriptomic analysis of the recombinant BCG demonstrated that *phoP-phoR* overexpression modified the expression of numerous proteins involved in lipid metabolism, potentially accounting for its enhanced immunogenicity [23]. Both LprO vaccine forms demonstrated comparable immunogenicity, eliciting significantly elevated levels of M. tb and LprO-specific CD8^+^ TNF-α^+^ T cell responses relative to the control group. These findings suggest that the anti-tubercular efficacy of the LprO single antigen is primarily mediated through the induction of CD8^+^ TNF-α^+^ T cell responses. Previous studies have established that CD8^+^ T cells possess multifunctional properties, enabling them to release cytokines or cytotoxic molecules upon activation, resulting in the apoptosis of target cells [24]. During M. tb infection, the host induces apoptosis in infected cells, encapsulating the bacteria within apoptotic bodies, which are subsequently phagocytosed by uninfected macrophages, thereby limiting bacterial dissemination [25]. Notably, prior research has shown that the M. tb lipoprotein Mpt83 can induce apoptosis in infected macrophages, suggesting that the mycobacterium lipoprotein, LprO, may exhibit similar capabilities [13,26].

Currently, 15 anti-tuberculosis vaccines are undergoing clinical trials, including nine subunit vaccines (encompassing viral vector, RNA, and protein-adjuvant platforms). Of these, all but two RNA vaccines (BNT164a1 and BNT164b1), for which the antigenic details remain undisclosed, incorporate multiple antigens. In this study, the DNA vaccine targeting tuberculosis contained only the LprO antigen, potentially limiting its protective efficacy. Given the resilience of M. tb, which is either eradicated by the host immune system or contained within granulomas leading to latent infection, incomplete clearance or control can result in active disease progression [27]. Future research should explore the integration of additional antigens to construct fusion proteins capable of eliciting a broader anti-tuberculosis immune response. Moreover, alternative subunit vaccine formats, such as protein–adjuvant vaccines, merit further investigation. For example, studies suggest that the protein–adjuvant form of the mycobacterium lipoprotein LppX induces higher IFN-γ levels compared to its DNA vaccine counterpart [14,28].

## 5. Conclusions

Our findings indicate that the LprO protein is a highly viable target for anti-tuberculosis vaccine development. Both DNA and recombinant BCG vaccines utilizing LprO as an antigen demonstrate substantial protective efficacy. The BCG Japan::pNBV1-*lprO* vaccine exhibits a strong safety profile in cellular assays and SCID mice, elicits a robust Th1-type immune response, and displays considerable immunogenicity, positioning it as a promising candidate for anti-tuberculosis vaccination.

## Figures and Tables

**Table 1:** Tuberculosis vaccines using lipoproteins as antigens.

Protein	Physiological Function	Vaccine Types	Vaccine Efficacy	Reference
LprG	Immunosuppression: Inhibition of Phagosome–Lysosome Fusion	M. tbΔ*lprG* Attenuated Live Vaccine	Comparable to BCG.	[6]
LpqS	Associated with Latent Mycobacterium Tuberculosis Infection	M. tbΔ*lpqS* Attenuated Live Vaccine	The protective effect in guinea pigs is superior to that of BCG.	[10]
LppZ	Highly Immunogenic	Protein Vaccine	Comparable to BCG.	[12]
RpfB	Associated with the Dormancy and Resuscitation of Mycobacteria	Protein Vaccine	In mice model, the lung and spleen bacterial loads are reduced by 2 log_10_ and 1 log_10_, respectively.	[11]
Mpt83	Promotes Apoptosis of Infected Macrophages	Protein and DNA Vaccine	In mice model, the lung and spleen bacterial loads are reduced by 1 log_10_ and 0.5 log_10_, respectively.	[13]
LppX	Associated with Mycobacterial Virulence	Protein Vaccine	The protein vaccine can elicit a strong IFN-γ response.	[14]
LpqH	Associated with Host Immunosuppression	LC3–LpqH Fusion Protein Vaccine	Reduces the bacterial load in the lungs and spleen of mice to some extent.	[15]
PstS-3	Phosphate Transport Receptor	PstS-3 & Ag85A Fusion DNA Vaccine; DNA vaccine	In mice model, the lung and spleen bacterial loads are reduced by 0.81 log_10_ and 0.6 log_10_, respectively; reduction of 1.5 log_10_ in the lungs.	[9]

## Data Availability

The original contributions presented in this study are included in the article or Supplementary Materials. Further inquiries can be directed to the corresponding author.

## Supplementary Materials

The following supporting information can be downloaded at: https://www.mdpi.com/article/10.3390/vaccines13040400/s1, Figure S1. B-cell Epitope Prediction Results of LprO Protein Based on the IEDB Database; Figure S2. 3D Structure of LprO Protein Predicted by AlphaFold 3; Figure S3. Successful expression of the LprO protein was achieved in Escherichia coli BL21 by induction with 0.5 mM isopropyl β-D-thiogalactoside (IPTG) for 20 hours at 16 °C; Figure S4. BALB/c mice were immunized subcutaneously with either BCG Japan::pNBV1 or BCG Japan::pNBV1-*lprO*, with ~10^6^ CFU per mouse; Figure S5. Gating strategies for T cell flow cytometry analysis; Figure S6. Bacterial load in the spleens of C57BL/6J mice in the mouse-M. tb infection model; Table S1. Excel file. Summary of CD4+ T cell and CD8+ T cell epitope predictions; Table S2. A peptide containing both T-cell and B-cell epitopes; Table S3. Primers.
