# Supplementary material for: DNA Subunit Vaccine and Recombinant BCG Based on Mycobacterial Lipoprotein LprO Enhance Anti-Tuberculosis Protection in the Lungs of Mice
Source: Vaccines (Basel). 2025 Apr 11;13(4):400. doi: 10.3390/vaccines13040400 (PMC12031346; doi:10.3390/vaccines13040400)
Supplement: Supplementary file 1 [file vaccines-13-00400-s001.zip › Supplementary Figures & Tables.pdf]

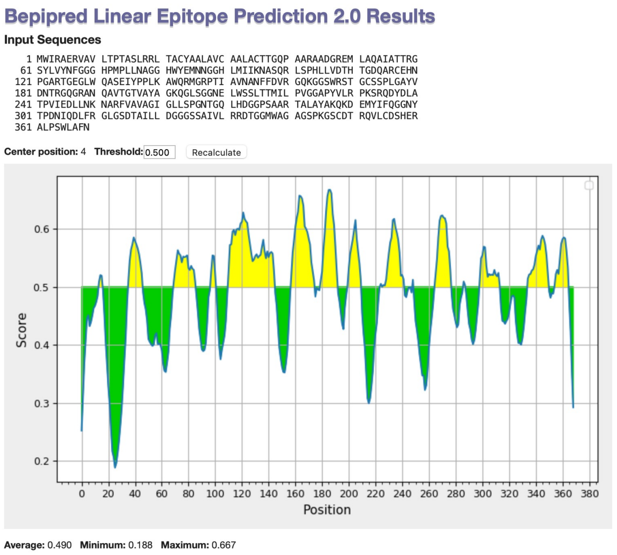

No.	Start	End	Peptide	Length
1	14	16	TAS	3
2	36	46	TTGQPAARAAD	11
3	70	86	GHPMPLLNAGGHWYEMN	17
4	98	101	SQRL	4
5	111	145	TGDQARCEHNPGARTGEGLWQASEIYPPLKAWQRM	35
6	158	175	DVRGQKGGSWRSTGCSSP	18
7	180	192	VDNTRGQGRANQA	13
8	200	210	AGKQGLSGGNE	11
9	225	225	A	1
10	227	242	YVLRPKSRQDYDLATP	16
11	249	249	N	1
12	266	277	GNTGQLHDGGPS	12
13	287	288	KQ	2
14	299	314	NYTPDNIQDLFRGLGS	16
15	335	350	GGMWAGAGSPKGSCDT	16
16	355	365	CDSHERALPSW	1

Figure S1. B-cell Epitope Prediction Results of LprO Protein Based on the IEDB Database

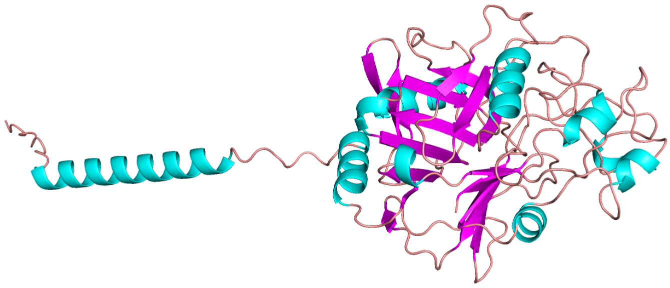

Figure S2. 3D Structure of LprO Protein Predicted by AlphaFold 3

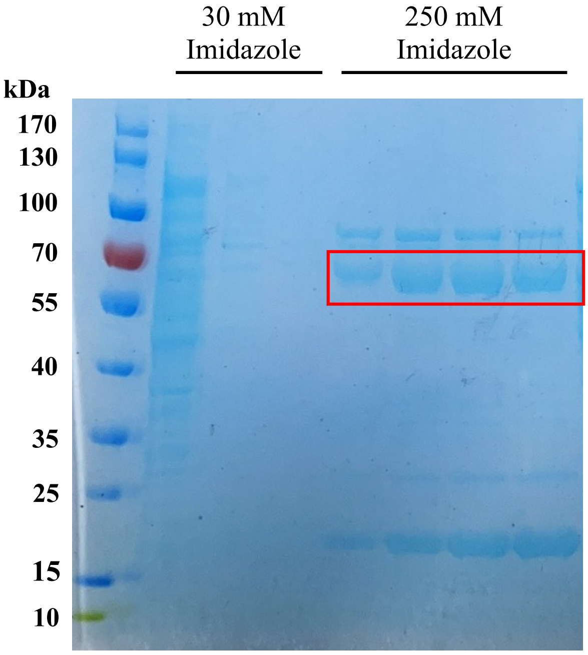

Figure S3. Successful expression of the LprO protein was achieved in *Escherichia coli* BL21 by induction with 0.5 mM isopropyl β-D-thiogalactoside (IPTG) for 20 hours at 16°C.

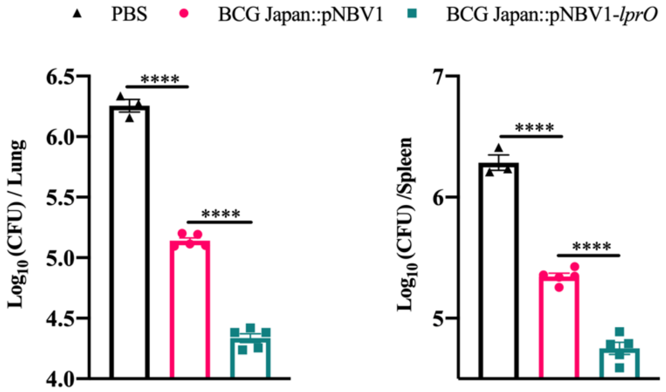

Figure S4. BALB/c mice were immunized subcutaneously with either BCG Japan::pNBV1 or BCG Japan::pNBV1-*lprO*, with ~10^6 CFU per mouse. Eight weeks post-immunization, mice were infected with ~10^7 CFU of BCG Pasteur via tail vein injection. Bacterial loads in the lungs and spleens were assessed three weeks post-infection. Bacterial load in the lungs and spleens of mice in the mouse-BCG Pasteur infection model (n=6 per group). Data are presented as mean ± SD. Statistical analysis was performed using ordinary one-way ANOVA with multiple comparisons, ****: $p < 0.0001$, ***: $p < 0.002$.

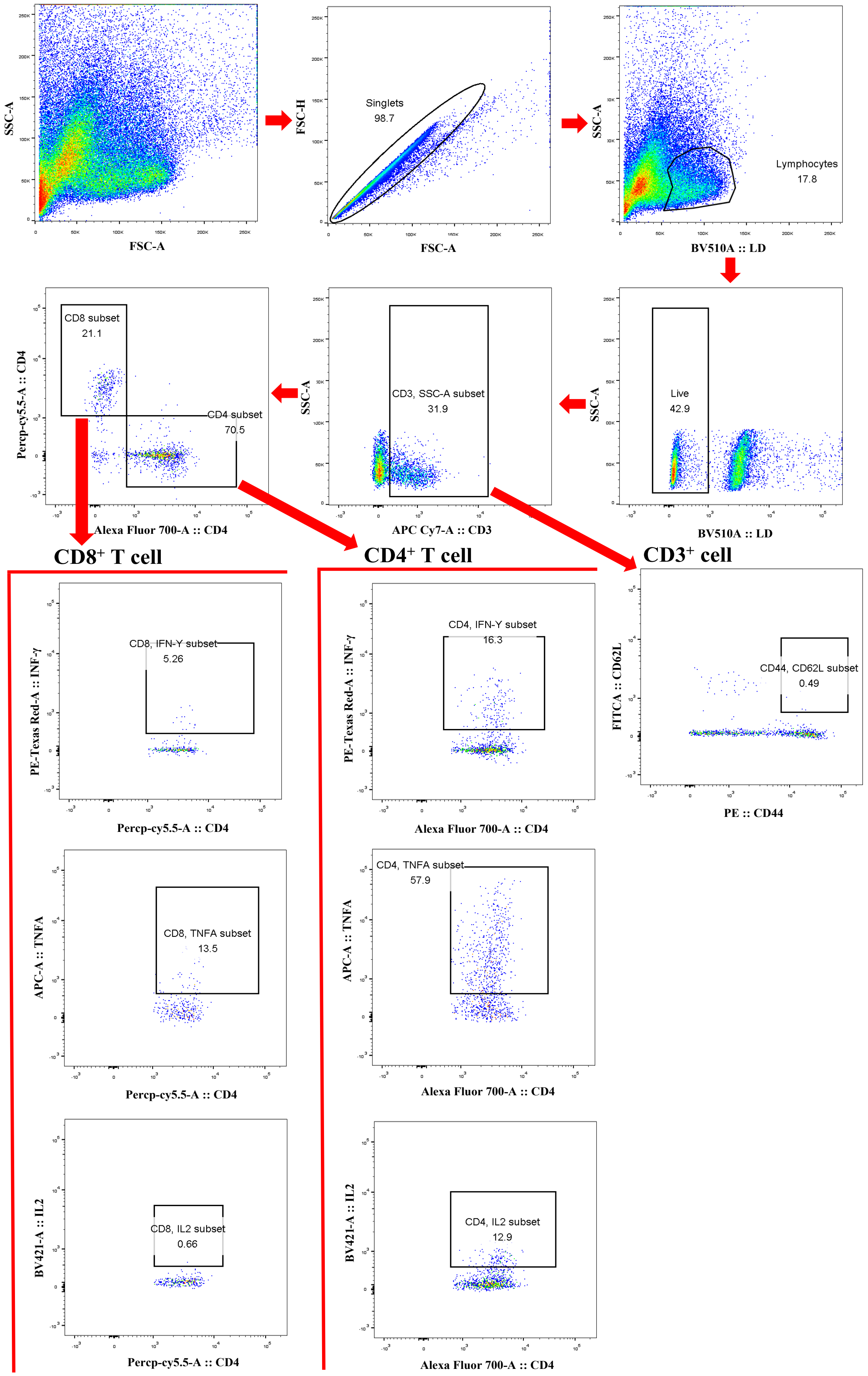

Figure S5. Gating strategies for T cell flow cytometry analysis.

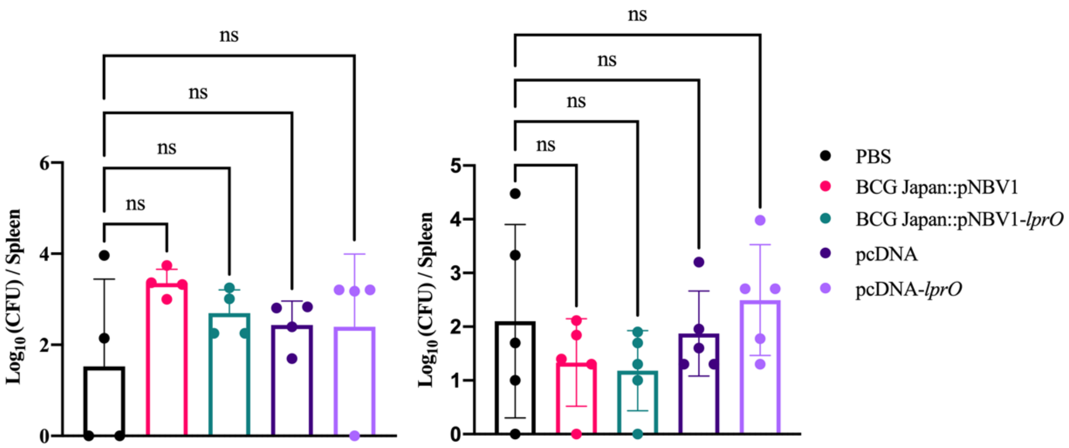

Figure S6. Bacterial load in the spleens of C57BL/6J mice in the mouse-M. tb infection model. The left graph displays results from the first round of experiments (n=4 per group), where the average bacterial load in the lungs on day 1 post-infection was 128 CFU; and the right panel shows results from the second round of experiments (n=5 per group), where the average bacterial load in the lungs on day 1 post-infection was 358 CFU. Data are presented as mean ± SD. Statistical analysis was performed using two-way ANOVA, with comparisons made against the PBS group, NS indicates no significance, $p > 0.05$.

Table S1. Excel file. Summary of CD4$^+$ T cell and CD8$^+$ T cell epitope predictions.

Table S2. A peptide containing both T-cell and B-cell epitopes

**Peptide**	**Sequence**	**Amino acids**
1	GHPMPL	6
2	GGHWYEMNN	9
3	RSTGCSSP	8
4	AGKQGLSGGNE	11
5	YVLRPKSRQDYDLATP	16
6	GNTGQLHDGGPS	12
7	NYTPDNIQD	9
8	GMWAGAGSP	9

Table S3. Primers

Name	Sequence	Function
pcDNA-*lprO*-F	ccc**aagctt**GCCACCATGgccgacgggcgcgagatg	Construction of pcDNA-*lprO*
pcDNA-*lprO*-R	ccg**gaattc**ttacttgtcgtcatcgtctttgtagtcgttgaacgccagccagctggg	Construction of pcDNA-*lprO*
sigA-F	acgcaaggacgccgaactca	qRT-PCR
sigA-R	gcaggttggcttccagcagatg	qRT-PCR
lprO-F	Gttgctgtgctgacaccgactg	qRT-PCR
lprO-R	Tgaccgccgccgaagttgta	qRT-PCR
pET-28a-*lprO*-F	ccggaattcatggccgacgggcgcgagat	Construction of pET-28a-*lprO*
pET-28a-*lprO*-R	cccaagcttttagttgaacgccagccagctggg	Construction of pET-28a-*lprO*
